# Unveiling the Gut Microbiota: How Dietary Habits Shape Health Through Microbiome Modulation

**DOI:** 10.3390/nu17101650

**Published:** 2025-05-12

**Authors:** Xi Wang

**Affiliations:** Department of Occupational and Environmental Health, School of Public Health, Xi’an Jiaotong University Health Science Center, Xi’an 710061, China; wn18andlife@xjtu.edu.cn

This Editorial provides an overview of the Special Issue “Dietary Habit, Gut Microbiome, and Human Health” which was recently published in *Nutrients*.

The intricate relationship between dietary habits and human health, mediated by the gut microbiome, has become a focal point of research in recent years [1,2]. As a complex and dynamically evolving ecosystem, the gut microbiome plays a critical role in host metabolism, immunity, and neurological function [3,4]. Diet acts as a primary modulator of gut microbiota composition, which subsequently influences the development and progression of various diseases [2]. This Special Issue, “Dietary Habit, Gut Microbiome, and Human Health”, compiles a series of cutting-edge articles that explore the interplay between diet, the gut microbiome, and human health. The nine included studies cover a broad spectrum of topics, ranging from the impact of specific dietary components on the gut microbiota to the therapeutic potential of dietary interventions in the prevention and management of diseases such as obesity, diabetes, autoimmune thyroid disease, cognitive impairments and endemic osteoarthritis. These articles not only provide novel insights into the mechanisms underlying diet–microbiome interactions but also underscore the potential of targeting the gut microbiome to facilitate disease prevention and treatment, thereby offering valuable guidance for future research and clinical practice in this domain.

Hernandez et al. investigate the effects of time-restricted feeding on cognitive function and gut microbiome composition in aging rats. Comparing time-restricted feeding with a ketogenic diet and a calorically matched control diet, they demonstrate that time-restricted feeding enhances cognitive performance irrespective of macronutrient composition. Furthermore, they identify significant differences in gut microbiome diversity and composition among the diet groups, and reveal that an abundance of Allobaculum is linked to cognitive task performance (Contributor 1). These findings suggest that time-restricted feeding may be a viable alternative to caloric restriction for improving cognitive and physical health in later life. This study also highlights the gut–brain axis as a promising therapeutic target for addressing cognitive dysfunction.

Wu et al. investigate alterations in the gut microbiota in rats resulting from selenium (Se) deficiency and exposure to T-2 toxin. Their study seeks to elucidate the potential implications of these factors with regard to Kashin–Beck disease (KBD). Their findings indicate that Se deficiency and T-2 toxin exposure induce significant changes in the gut microbiota of rats. In their research, at the phylum level, Firmicutes predominated in both the Se-deficient (SD) group and the T-2 toxin exposure group, whereas the proportion of Bacteroidetes was reduced in the SD group compared to the T-2 group. At the genus level, Ruminococcus_1 and Ruminococcaceae_UCG-005 exhibited higher relative abundance in the SD group, while Lactobacillus and Ruminococcaceae_UCG-005 were more prevalent in the T-2 group. This study underscores the distinct modifications in gut microbiota induced by Se deficiency and T-2 toxin, offering valuable insights into the potential role of gut microbiota in the pathogenesis of KBD (Contributor 2). It highlights the need for further investigation into the complex interactions between environmental factors, gut microbiota, and joint health.

Fagunwa et al. explore the effects of dietary sodium on gut microbiota, revealing that a high-sodium diet (HSD) significantly alters the microbiota by decreasing the presence of Bacteroides and increasing the prevalence of Prevotella compared to a low-sodium diet (LSD). Their study suggests that the Bacteroides/Prevotella (B/P) ratio is a more precise marker for gut microbiota changes than the Firmicutes/Bacteroidetes (F/B) ratio, due to its clearer differentiation between sodium intake levels. They also identify sodium reabsorption genes in the epithelial sodium channel of the guts of people who eat an HSD as opposed to an LSD (Contributor 3). These findings underscore the B/P ratio’s potential as an indicator of dietary sodium intake and its effects on gut health, offering new insights and directions for future research on the gut microbiome’s role in health.

Duysburgh et al. investigate the prebiotic effects of baobab fiber (BF) and Arabic gum (AG), both individually and in combination, and demonstrate that BF and AG significantly influence the composition and metabolic activity of the gut microbiota in both the proximal and distal colon. Notable findings within this work include an increase in Bifidobacteriaceae and Faecalibacterium prausnitzii populations, enhanced production of short-chain fatty acids (SCFAs), and a reduction in branched-chain fatty acids (BCFAs) and ammonium levels. The co-supplementation of BF and AG at a lower dosage exerts synergistic prebiotic effects, evidenced by biological activity throughout the entire colon, increased abundance of Akkermansiaceae and Christensenellaceae in the distal colon, and elevated levels of spermidine and other metabolites of interest (Contributor 4). This research underscores the potential of BF and AG co-supplementation as a promising prebiotic strategy for enhancing gut health.

The review conducted by Chen et al. provides an in-depth analysis of the complex interplay between dietary patterns, gut microbiota, and athletic performance. It elucidates the pivotal role of gut microbiota in human health, particularly its influence on metabolism and immune function. The authors underscore the significant impact of both training and nutritional strategies on athletic performance, examining how various dietary patterns modulate the gut microbiota, which subsequently affects sports performance (Contributor 5). The review details the primary functions of the gut microbiota and its association with exercise, indicating that athletes possess distinct gut microbiota compositions compared to sedentary individuals, characterized by greater diversity and an increased abundance of beneficial species. Ultimately, the review posits that specific dietary patterns can enhance sports performance by modulating the gut microbiota.

Wang et al. examine the effects of integrating exercise with konjac glucomannan (KGM) on antibiotic-induced dysbiosis of the gut microbiota in mice. Their findings indicate that this combined intervention is more effective than individual interventions in restoring alterations in gut microbiota composition and diversity caused by antibiotic treatment. Furthermore, in their work, a combination of exercise and KGM significantly enhances microbial purine metabolic pathways. This study underscores the potential of employing a combination of exercise and KGM as a promising strategy to mitigate the adverse effects of antibiotics on the gut microbiome (Contributor 6). Nonetheless, further research is warranted to determine the optimal timing and intensity of exercise interventions. This study offers valuable insights for the development of strategies aimed at preventing and managing gut microbiota dysbiosis.

Yun et al. investigate the impact of Lactobacillus plantarum P111 and Bifidobacterium longum P121 on obesity and depression or cognitive impairment-like behavior induced by a high-fat diet (HFD) in mice. Their findings indicate that these probiotics, whether administered individually or in combination, can mitigate HFD-induced body weight gain, liver steatosis, and depression or cognitive impairment-like behavior. The results demonstrate that oral administration of P111 or P121 significantly reduces HFD-induced weight gain and enhances lipid profiles in both the blood and liver. Moreover, these probiotics downregulate NF-κB activation and TNF-α expression in the liver and colon while concurrently upregulating AMPK activation. They also attenuate HFD-induced depression/cognitive impairment-like behavior and hippocampal NF-κB activation, leading to an increase in the hippocampal BDNF-positive cell population and BDNF levels (Contributor 7). Notably, the combined administration of P111 and P121 (LpBl) exhibits a more pronounced effect in ameliorating body weight gain, liver steatosis, and depression or cognitive impairment-like behavior. This study underscores the potential of P111 and P121 as promising candidates for the prevention and treatment of these conditions.

Zhang et al. investigate the causal relationship between gut microbiota and autoimmune thyroid disease (AITD) through the application of a Mendelian randomization approach. Following an examination of 119 gut microbiota and 9 metabolites, their research identifies a causal association between 3-indoleglyoxylic acid levels and the risk of AITD, implying that metabolites derived from gut microbiota may play a role in the pathogenesis of AITD. Additionally, this study underscores the potential causal effects of Ruminococcus torques on Graves’ disease and taurocholic acid on Hashimoto’s thyroiditis (Contributor 8). In summary, this research offers novel insights into the influence of gut microbiota on AITD, thereby laying the groundwork for future investigations and potential therapeutic interventions.

Type 2 diabetes (T2D), a chronic metabolic disorder with extensive health implications, has been increasingly associated with gut microbiota dysbiosis. The review article “Targeting the Gut Microbiota for Prevention and Management of Type 2 Diabetes” provides valuable insights into this intricate relationship. The authors conduct a comprehensive analysis of how alterations in the gut microbiota, including imbalances in bacterial species and changes in metabolite production, contribute to the pathogenesis of T2D (Contributor 9). They emphasize the potential of modulating the gut microbiota through nutritional interventions, such as specific diets and supplements, as well as physical exercise, to restore microbial homeostasis and enhance glycemic control. This review improved our understanding of the gut microbiota–T2D connection and its implications for the development of novel therapeutic strategies for T2D management.

This Editorial summarizes the nine articles that comprise this Special Issue, emphasizing the crucial link between dietary patterns, gut microbiota, and human health. The findings deepen our understanding of the mechanisms involved in these processes and suggest promising strategies for disease prevention and treatment, paving the way for future research and practical applications in this dynamic field.
